# The Maidstone Epidemic

**Published:** 1898-02-05

**Authors:** 


### THE MAIDSTONE EPIDEMIC.

The Local Government Board inquiry into the recent outbreak of typhoid fever was opened on January 31st at Maidstone, before Mr. J. S. Davy, Mr. G. W. Wilcocks, and Dr. Theodore Thomson. Mr. Percy Adams, deputy medical officer of health, who was acting at the time the epidemic commenced, and Mr. Matthew Adams, the medical officer of health, were examined, and gave the history of the outbreak, which they attributed to specific pollution of the Farleigh section of the Maidstone water supply, and more especially of the Tutsham springs. They had, however, not been able to ascertain that any of the hop pickers who had been encamped in the neighbourhood had suffered from typhoid fever. The drainage only affected the epidemic by causing secondary cases.

